# Pomegranate‐Derived Exosome‐Like Nanovesicles Containing Ellagic Acid Alleviate Gut Leakage and Liver Injury in MASLD

**DOI:** 10.1002/fsn3.70088

**Published:** 2025-04-10

**Authors:** Ji‐Su Kim, Byoung‐Joon Song, Young‐Eun Cho

**Affiliations:** ^1^ Department of Food and Nutrition Andong National University Andong South Korea; ^2^ Section of Molecular Pharmacology and Toxicology National Institute on Alcohol Abuse and Alcoholism Bethesda Maryland USA

**Keywords:** ellagic acid, leaky gut, liver fibrosis, metabolic dysfunction‐associated fatty liver disease, plant‐derived exosome‐like nanovesicles, pomegranate‐derived exosome‐like nanovesicles

## Abstract

Exosome‐like nanovesicles derived from plants (PENs) harbor a spectrum of bioactive compounds, including proteins, lipids, nucleic acids (such as miRNAs and mRNAs), offering therapeutic advantages for a variety of diseases. This investigation assesses the utility of pomegranate‐derived exosome‐like nanovesicles (PNVs) in both preventing and treating complications such as liver damage and increased intestinal permeability found in Metabolic Dysfunction‐Associated Steatotic Liver Disease (MASLD). Utilizing Transmission Electron Microscopy (TEM) and Nanoparticle Tracking Analysis (NTA), we successfully isolated PNVs and characterized their structural properties. Upon administration in a live model, these nanovesicles were efficiently distributed to critical organs, including the liver and intestines, demonstrating biocompatibility by avoiding toxic effects. Remarkably, these vesicles were enriched with ellagic acid, known for its strong antioxidant capabilities. In a controlled MASLD mouse study, treatment with PNVs significantly lowered serum endotoxin levels, reinforced intestinal barrier functions, and altered the gut microbiota profile favorably. Furthermore, the application of PNVs reduced oxidative stress and the presence of fibrosis markers in the liver to normal levels. These results indicate that PNVs could be a viable option for addressing MASLD, enhancing intestinal integrity, reducing liver injury, and diminishing fibrosis through the modulation of the gut‐liver axis.

## Graphical abstract

Pomegranate‐derived exosome‐like nanovesicles (PNVs) were successfully isolated and characterized for their size, concentration, and morphology. PNVs rich in ellagic acid (EA) can be utilized as a therapeutic agent to treat liver damage resulting from metabolic dysfunction‐associated fatty liver disease (MASLD). Administration of PNVs containing EA led to decreased serum endotoxin levels and the restoration of intestinal barrier proteins.

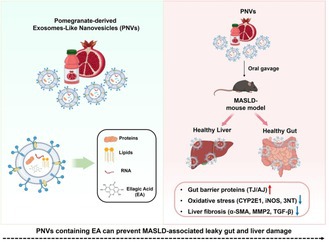

## 1 Introduction

Metabolic dysfunction‐associated steatotic liver disease (MASLD) is a prevalent condition closely linked to metabolic disorders such as obesity and Type 2 diabetes. In its advanced stages, MASLD progresses to steatosis, steatohepatitis (hepatic inflammation), fibrosis/cirrhosis, and, in severe cases, malignant tumors. This disease is often associated with gut microbiota imbalances and their related metabolites, which influence immune system function (Libman et al. 2019; Schwärzler et al. 2024). The gut microbiome plays a crucial role in maintaining energy homeostasis, metabolizing various compounds, and regulating immune responses through complex molecular mechanisms. Notably, dietary diversity, particularly the composition of plant‐based foods and supplements, has been shown to significantly alter the composition and abundance of gut microbiota in a relatively short period (Carbonero 2021; Li et al. 2021; Pferschy‐Wenzig et al. 2022; Puhlmann and de Vos 2022). Individuals who consume excessive amounts of high‐fat diets (HFDs), including cholesterol‐rich fast food, while neglecting plant‐based nutrition, are more prone to gut dysbiosis, which may contribute to immune dysregulation and barrier dysfunction (Cox et al. 2015; David et al. 2014; Pindjakova et al. 2017). Despite the significant burden MASLD places on public health, only one drug has been officially approved for its treatment. However, lifestyle interventions, including regular exercise, a nutritionally balanced diet, and the use of dietary supplements, continue to be promising strategies for the prevention and management of MASLD (Ganakumar et al. 2025; Song et al. 2024; Younossi et al. 2021).

Plant‐derived exosome‐like nanovesicles (PENs) are small, bilayer membrane‐enclosed vesicles secreted by plant cells, recognized as promising natural nanocarriers for next‐generation drug delivery (Ran et al. 2024). These PENs are composed of various bioactive molecules, including proteins, functional lipids, RNA, mRNA, and miRNA. With a size range of approximately 50–300 nm, PENs are considered biocompatible, sustainable, and non‐toxic nanocarriers. Compared to mammalian‐derived extracellular vesicles, PENs offer several advantages, such as cost‐effectiveness, mass production, high stability, and efficient intercellular communication and interaction (Liu et al. 2024; Madhan et al. 2024; Pan et al. 2024; Wang et al. 2024a, 2024b). Recently, our laboratory demonstrated the alleviating effects of PENs against various diseases such as inflammatory bowel disease (IBD), osteoporosis, alcohol‐associated fatty liver disease (ALD), and anti‐cancer (Eom et al. 2022; Hwang et al. 2023; Kim et al. 2024, 2023a, 2023b). Moreover, studies exploring the health benefits of PENs have been steadily increasing in recent years (Alfieri et al. 2021; Suharta et al. 2021).

**FIGURE 1:**
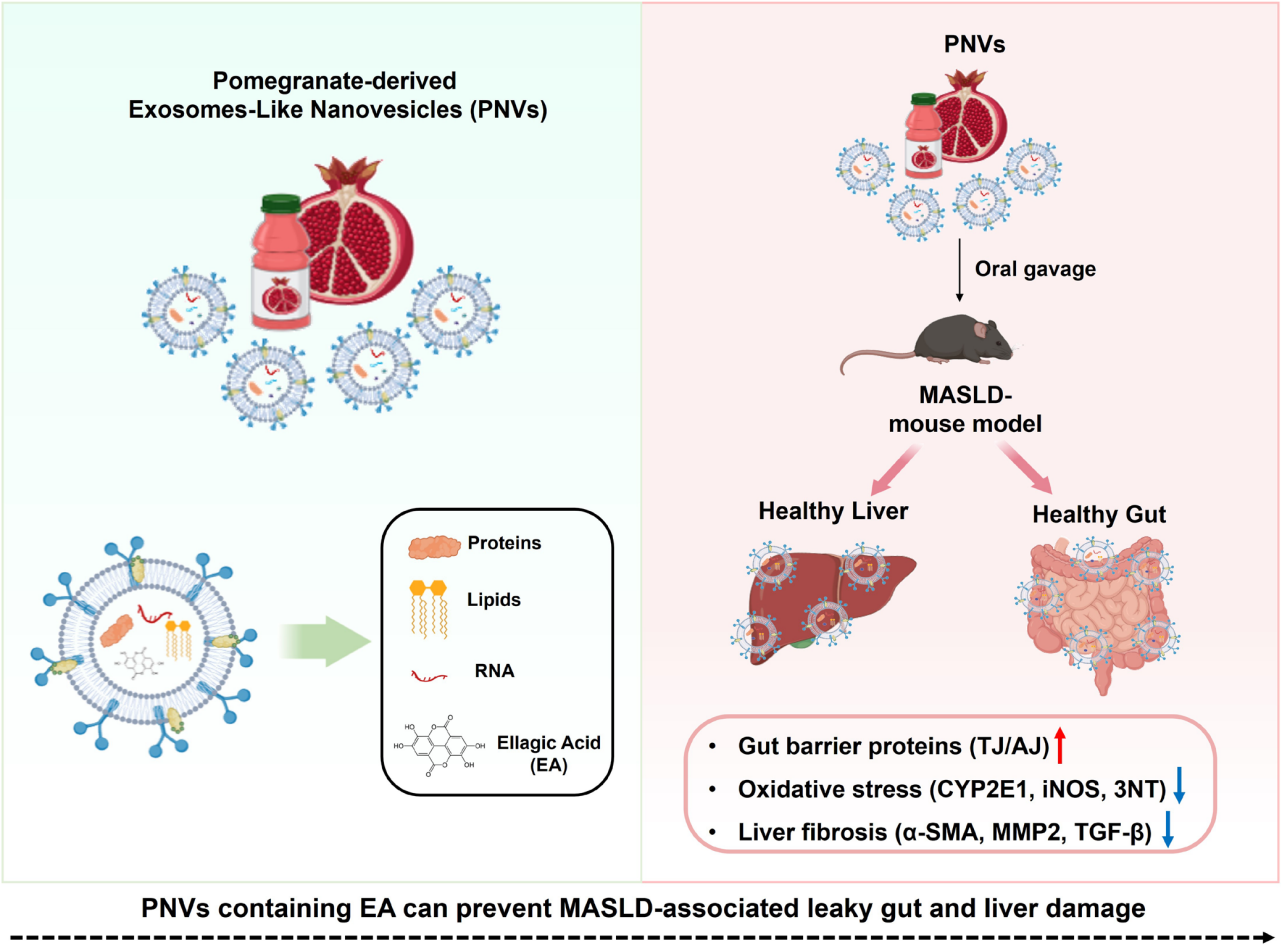
Schematic diagram to describe isolation, administration, and protective effects of PNVs against leaky gut and liver damage in the WD‐mediated MASLD model.

The pomegranate ( *Punica granatum* ) is a widely consumed fruit known for its rich content of bioactive compounds, including polyphenols, flavonoids, tannins, and ellagic acid. All edible parts of the pomegranate—seeds, juice, and peel have been reported to provide various health benefits (Ismail et al. 2012; Kim et al. 2023a, 2023b; Siddiqui et al. 2024). In particular, pomegranates are a valuable source of EA, a bioactive compound with potential applications in managing both mild and chronic diseases (Tamborlin et al. 2020). Recent studies highlight that pomegranates exhibit anti‐cancer (Kim et al. 2024), antioxidant (Tavakoli et al. 2024), anti‐inflammatory (Pierdomenico et al. 2024), anti‐diabetic (Gościniak et al. 2024), and liver‐protective properties (Kim et al. 2023a, 2023b). Pomegranate alleviated HFD‐induced obesity, insulin resistance, and hepatic steatosis in mice, along with the changes in the composition and abundance of gut microbiota (Song et al. 2022). In fact, PNVs are small extracellular vesicles that transport bioactive molecules, such as proteins, lipids, and nucleic acids, which can interact with and alter the composition, abundance, and function of the gut microbial community. However, there are few studies on the gut‐liver axis through the regulation of gut microbiota using PNVs, and no systematic study has been conducted on their beneficial mechanisms. Thus, this study aims to examine the protective effects of PNVs against HFD‐induced MASLD, which is accompanied by gut and liver injury. To the best of our knowledge, this is the first report to provide evidence that PNVs, particularly those rich in EA, can prevent gut barrier dysfunction and liver damage/fibrosis in a MASLD mouse model (Figure 1).

## 2 Materials and Methods

### 2.1 Animal Ethics and Treatment

All animal experiments were conducted with approval from the Andong University Institutional Animal Care and Use Committee (Approval No. 2021‐2‐0420‐01‐01) and followed the guidelines for animal testing at Andong University. All mice used in this study were obtained from Orient Bio Inc. (Seongnam‐si, Korea) and maintained under a 12 h light–dark cycle with unrestricted access to food and water. MASLD was induced by feeding Western‐style high fat (DooYeol Biotech, Seoul, Republic Korea). Animals were randomly assigned to four groups (*n* = 5–10 mice/group) as follows: (a) CON group: fed a standard chow; (b) WD group: fed a WD diet for 8 weeks; (c) WD + PNVs group were treated with oral administration of 1 mg/kg/day PNVs for 3 days or 5 weeks; and (d) WD + EA group were treated with oral administration of 60 mg/kg/day EA for 3 days or 5 weeks.

### 2.2 Isolation of Exosome‐Like Nanovesicles From Pomegranates

Fresh pomegranate juice was purchased from Georgia's Natural, Tbilisi, GA, USA. Nanovesicles were isolated according to the method developed in our laboratory (Eom et al. 2022; Hwang et al. 2023; Kim et al. 2023a, 2023b). Each exosome pellet was further characterized and dissolved in phosphate‐buffered saline (PBS). The protein concentration in the samples was measured using a BCA protein assay (Pierce, Rockford, IL, USA) (Cho et al. 2021).

### 2.3 Nano‐Size Confirmation

The particle size and concentration of PNVs were analyzed through nanoparticle tracking analysis (NTA) using the Nanosight NS300 system (NanoSight, Amesbury, UK). For the transmission electron microscope (TEM) analysis, PNV samples were filled into glow‐discharge copper grids coated with a continuous carbon film and stained with 0.75% uranyl formate before image scanning (Cho and Song 2018).

### 2.4 Quantification of EA by HPLC

The EA content was quantified by HPLC. The chromatographic conditions for EA are described in Table S1. After diluting the PNVs sample with ethanol 1:1, the sample mixture was extracted with ultrasonic waves for 10 min, centrifuged at 15,000 rpm, filtered through a 0.2 μm filter, and the EA content was analyzed by HPLC.

### 2.5 Oral Glucose Tolerance Test

After 5 weeks of PNVs treatment, blood was drawn from the tail of each mouse in all groups to measure basal glucose and insulin levels. For the oral glucose tolerance test (OGTT), mice were fasted overnight before receiving a single oral dose of glucose (150 mg per mouse). Blood samples were then collected at 0, 30, 60, 90, and 120 min after glucose administration. The blood glucose level of all samples was immediately measured using a G400 green doctor blood glucose meter (Mei et al. 2010).

### 2.6 In Vivo Uptake Assay of PNVs

The uptake of PNVs in mice was examined using an in vivo imaging system (IVIS; NEWTON 7.0, Vilber, Collégien, France). For fluorescence imaging, PNVs (1 mg/kg) were labeled by incubating them with 10 μL of DiR (1,1’‐Dioctadecyl‐3,3,3′,3’‐Tetramethylindotricarbocyanine iodide) for 30 min, followed by centrifugation at 100,000 × *g* for 1 h. The supernatant was discarded and the DiR‐labeled PNVs pellets were dissolved in PBS and orally administered to each mouse (*n* = 3 ~ 4/group). After 24 h, the indicated organs were removed and the digestion and absorption patterns of administered DiR‐labeled PNVs were evaluated in situ.

### 2.7 Histological Analysis

Liver and small intestine samples from mice in each group were fixed in neutral formalin and embedded in paraffin blocks. The tissue sections were then stained using hematoxylin and eosin (H and E) or Sirius Red solution (Kyungpook National University core lab) (Cho et al. 2017). Histological changes were evaluated under a light microscope (Cho et al. 2017).

### 2.8 Measurements of Serum Alanine Transaminase, Serum Endotoxin, and Hepatic Triglyceride Levels

The levels of serum alanine transaminase (ALT), aspartate transaminase (AST), and hepatic triglyceride (TG) were determined using the commercial assay kits such as the standard end‐point colorimetric assay kits (BioVision, Milpitas, CA, USA, and Asan Co. Ltd., Gimpo, Korea, respectively). Serum endotoxin levels were determined using the commercial detection kit (endpoint LAL Chromogenic Endotoxin Quantitation Kit; Thermo Fisher Scientific, Waltham, MA, USA) (Cho and Song 2018; Cho et al. 2018).

### 2.9 Immunoblot Analysis

Tissues and cells were homogenized in 1 × RIPA buffer using a homogenizer. The resulting lysates were subjected to SDS/PAGE for protein separation and then transferred onto nitrocellulose membranes. The nitrocellulose membranes were then incubated with the specific primary antibodies described in Table S2. After three separate cycles of washing steps, the nitrocellulose membranes were then incubated with the HRP‐conjugated secondary antibody before images were detected using a ECL solutions (Thermo Fisher). The intensities of immunoreactive target protein bands were quantified by densitometry using a chemiluminescence imaging system (FUSION SOLO S, Vilber, Collégien, France), with GAPDH serving as the loading control.

### 2.10 Proteomic Analysis of PNVs

The purified PNVs were sent to Protia Inc. for proteomic analysis. For proteomic analyses, PNVs proteins were extracted and subjected to enzymatic digestion, followed by analysis and identification by LC–MS/MS. Mascot software was used for library identification analysis. GO identifications of all identified proteins using the DAVID software were employed.

### 2.11 Microbial 16S Sequencing and Bioinformatics

Stool samples were aseptically obtained from the cecum of each mouse and immediately stored at −80°C. DNA was extracted using the Mag‐Bind Universal Pathogen DNA Kit (CJ Bioscience, Seoul, South Korea) according to the manufacturer's instructions. Sequencing of the bacterial 16S ribosomal RNA and subsequent bioinformatics analysis for each cecum sample was conducted at CJ Bioscience (https://www.cjbioscience.com/ngs).

### 2.12 Statistical Analysis

The data were presented as the mean ± standard error, and all statistical analyses were performed using SPSS for Windows 27.0 (SPSS Inc., Chicago, IL, USA). Statistical significance was assessed at the *p* < 0.05 level, and differences were evaluated using one‐way or two‐way ANOVA followed by Duncan's multiple range test post hoc analysis (Cho and Song 2018; Cho et al. 2018).

## 3 Results

### 3.1 Purification and Characterization of PNVs

**FIGURE 2:**
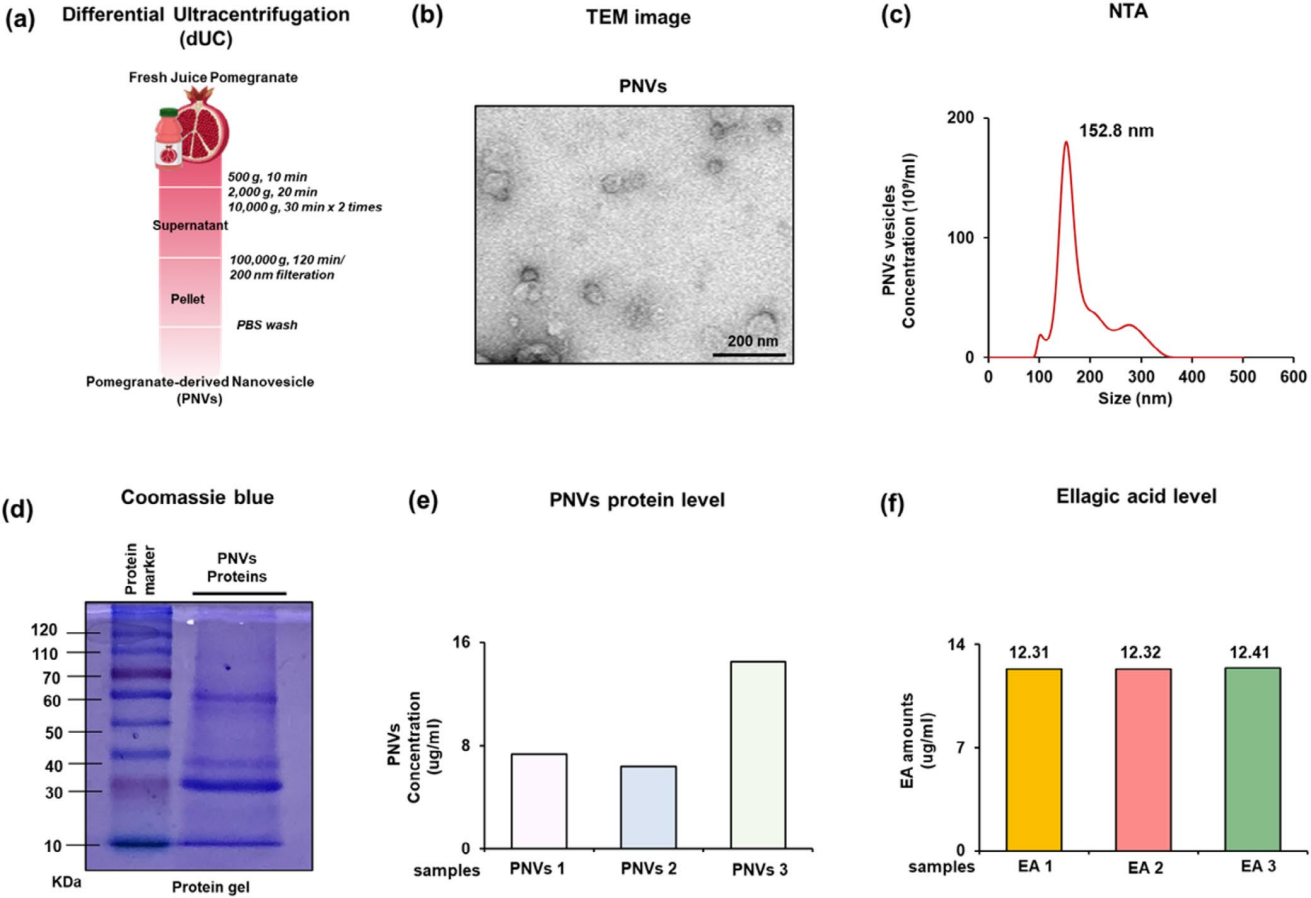
Isolation and characterization of PNVs. (a) Flowchart of differential ultracentrifugation procedures to isolate PNVs. (b) TEM image of PNVs. (c) Nanoparticle Tracking Analysis (NTA) of size and concentration of the isolated PNVs. (d) Pattern of SDS‐polyacrylamide gel electrophoresis of PNV proteins. The proteins extracted from PNVs were separated using SDS‐PAGE and stained with Coomassie blue dye. (e) Final concentrations of each of the PNV proteins of pomegranate. (f) HPLC analysis of each of ellagic acid (EA).

Exosome‐like nanovesicles (Figure 2a) were isolated from fresh pomegranate juice using a previously described purification technique (Kim et al. 2023a, 2023b). The morphology and size of PNVs were characterized by TEM analysis (Figure 2b). The lipid layer morphology was confirmed, and the size of PNVs was approximately 200–300 nm (Figure 2b). Average size distribution and concentration were measured to be 152.8 nm and 1.1 × 10^9^ particles/mL using NTA analysis, respectively (Figure 2c). Therefore, we confirmed that purified PNVs exhibit the shape and size of typical plant‐derived exosome‐like nanovesicles. The molecular sizes of major proteins in PNVs were in a range of 30–60 kDa (Figure 2d). The isolation of PNVs from 5 mL of pomegranate juice resulted in a protein concentration of 8–16 μg/mL (Figure 2e). Pomegranate juice is rich in bioactive substances with anti‐inflammatory and antioxidant properties, such as major ellagic acid (EA), polyphenols, and tannins (Tamborlin et al. 2020; Viuda‐Martos et al. 2010). Interestingly, large amounts of EA were consistently detected in three different batches of isolated PNVs (Figure 2f). As a result of high‐performance liquid chromatography‐photodiode array spectrum (HPLC) analysis, the peak of EA was very strong in PNVs (Figure S1). To investigate the profile of PNVs components, we performed proteomic analysis. Proteins related to chitinase, lipid transfer protein, and chalcone synthase were abundantly expressed in PNVs. In particular, gene ontology (GO) analysis revealed that the levels of gene expression of bifunctional inhibitor/plant lipid transfer protein/seed storage helical (Bifunc_inhib/LTP/seed_store), lipid transport, and lipid binding were high (Figure S2). This analysis suggests that PNVs containing various proteins and bioactive substances, including EA, can be an optimized drug delivery platform in suppressing MASLD mouse models.

### 3.2 In Vivo Distribution Uptake of PNVs

**FIGURE 3:**
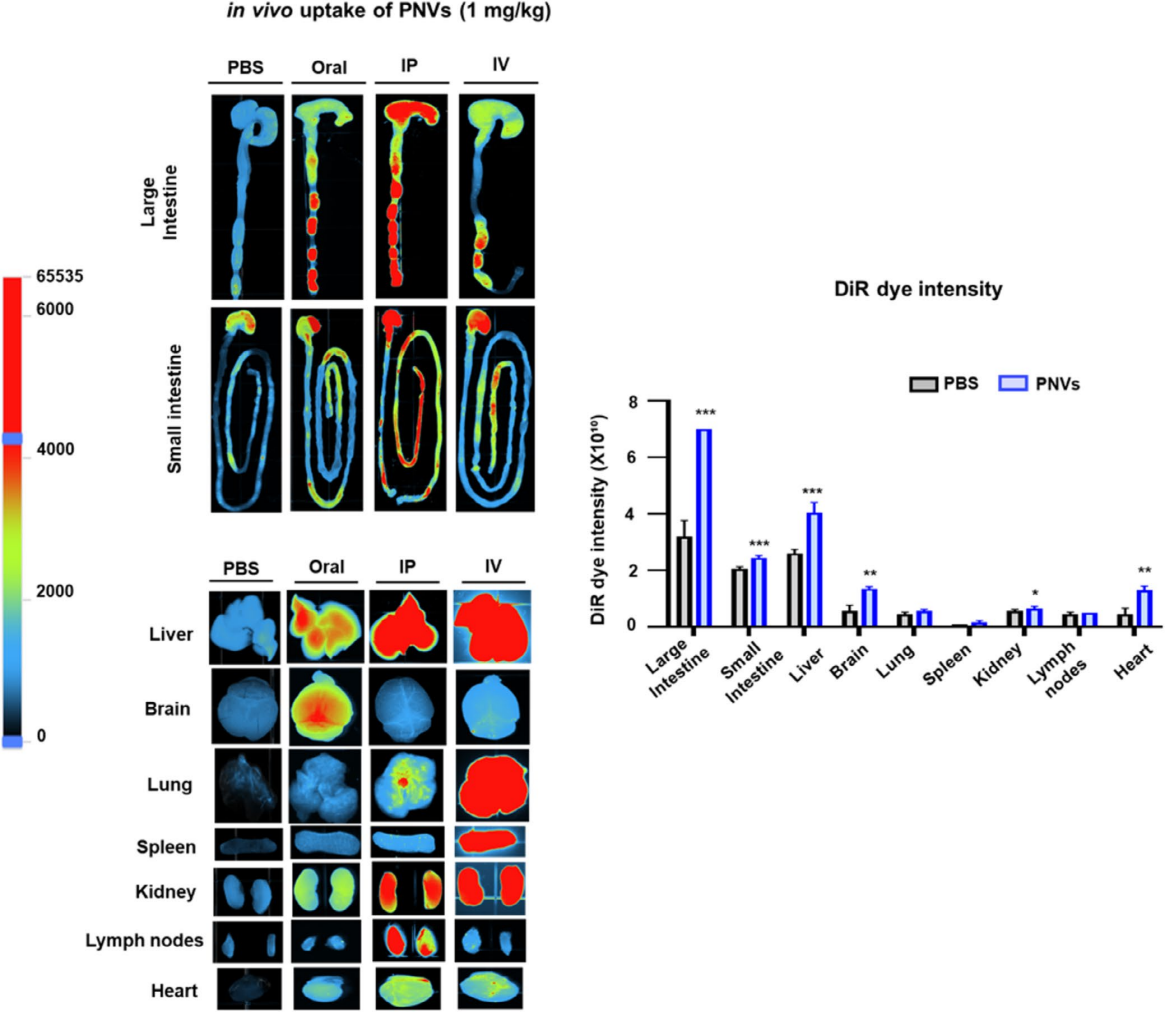
In vivo tissue distribution of PNVs. (a) At 24 h after oral, IP, or IV administration of PNVs, ex vivo images of DiR‐labeled PNVs in various tissues, including small intestine, large intestine, brain, lung, heart, liver, spleen, kidney, and lymph nodes, were acquired with an IVIS spectrum. The DiR dye intensity was calculated using the Living Image 3.1 software.

To study the biodistribution, male mice (*n* ≥ 3/group) were treated with DiR dye‐labeled PNVs (1 mg/kg) through oral, IP, or IV administration and imaged over 24 h (Figure 3). Image densities of several organs (e.g., colon, small intestine, liver, brain, lung, spleen, kidney, lymph nodes, and heart) were determined after oral, IP, or IV administration of DiR dye‐labeled PNVs (Figure 3). High amounts of orally administered DiR‐labeled PNVs were detected in the colon, small intestine, liver, and brain, although their intensities are generally much weaker than those of IV injection and lower than those of IP administration. Therefore, these results confirmed that PNVs were nano‐sized nanovesicles that were selectively distributed to certain organs, such as the liver, brain, and intestine.

### 3.3 PNVs Attenuated the Levels of Elevated TG and Oxidative Stress Proteins in MASLD Mice

**FIGURE 4:**
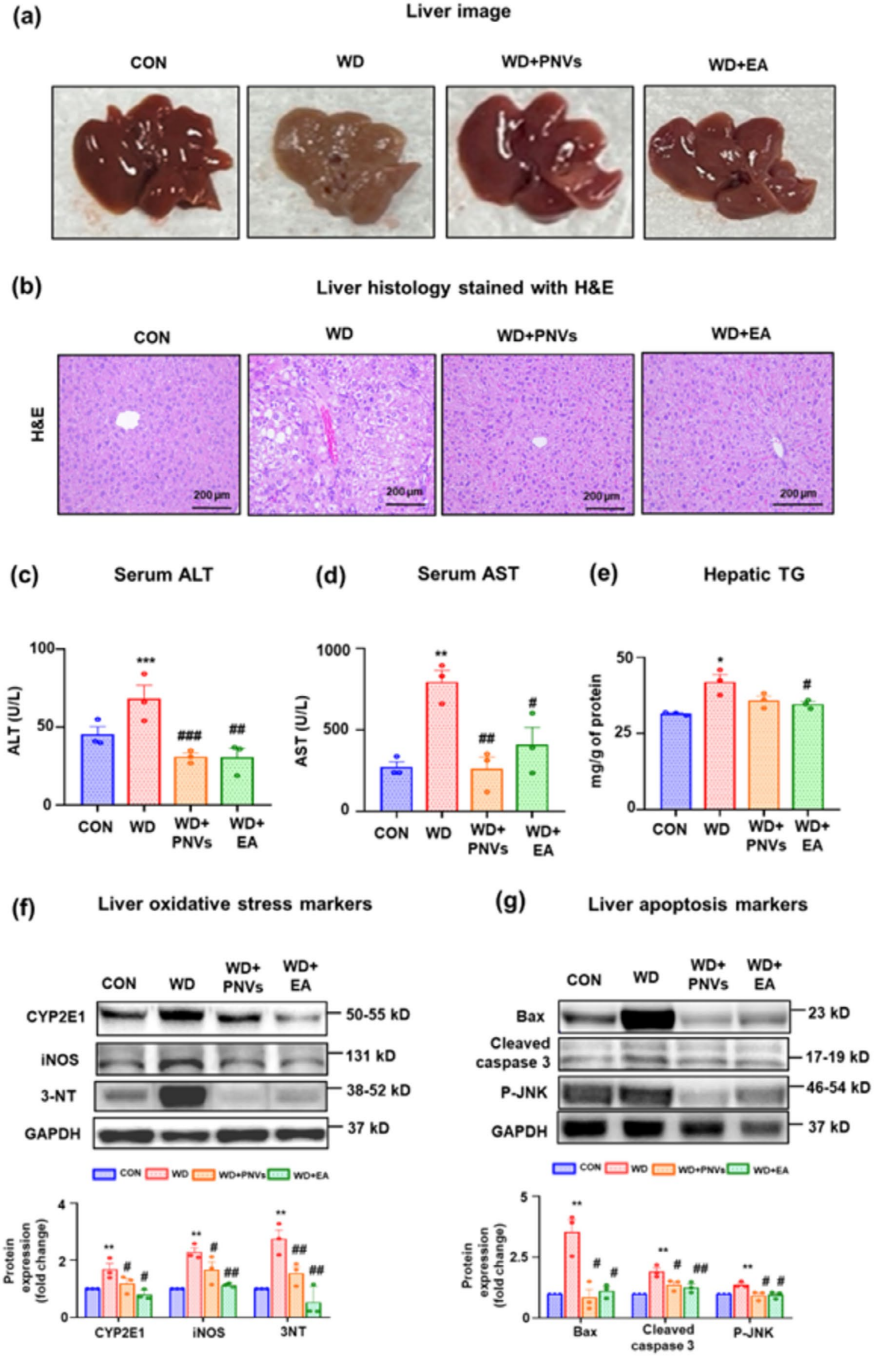
PNVs treatment attenuated WD‐induced liver injury in MASLD mice. (a) Representative visual liver image for CON, WD, WD + 1 mg/kg PNVs, or WD + 60 mg/kg EA group, as indicated. (b) Representative H and E‐stained liver sections for CON, WD, WD + 1 mg/kg PNVs, or WD + 60 mg/kg EA group, as indicated. The levels of (c) Serum ALT, (d) Serum AST, and (e) Hepatic TG are shown. Immunoblot analyses for (f) Oxidative stress markers (CYP2E1, iNOS, 3NT), (g) Apoptosis markers (Bax, Cleaved caspase 3, P‐JNK) for the indicated groups are presented. Densitometric analysis of immunoblot for each protein relative to the GAPDH loading control is shown. Each immunoblot experiment was repeated at least three times independently, and consistent results were observed. *p < 0.05, **p < 0.01, ***p < 0.001 between CON and WD groups; #*p* < 0.05, ##*p* < 0.01, ###*p* < 0.001 between WD and WD + 1 mg/kg PNVs; #*p* < 0.05, ##*p* < 0.01, ###*p* < 0.001 between WD and WD + 60 mg/kg EA group. The significance of mean values for each group was determined using the Student's *T*‐test.

Liver weights in MASLD with and without PNVs or EA treatment did not significantly change from those of the control group (Figure S3a). Visual images confirmed fatty liver damage in the WD groups compared to the control group. However, the oral administration of PNVs and EA alleviated these abnormalities in the liver (Figure 4a). H and E‐stained liver histological slides showed elevated hepatic lipid droplets and inflammation foci in the WD group compared to the control group, and this elevation was attenuated by the administration of PNVs and EA (Figure 4b). Serum ALT and AST levels were significantly elevated in the WD group compared to the control group, and these elevations were considerably decreased by the administration of both PNVs and EA (Figure 4c,d). Additionally, the hepatic triglyceride (TG) level was significantly increased in the WD group, and the administration of PNVs or EA significantly decreased the elevated TG levels (Figure 4e). Furthermore, fasting glucose levels determined by oral glucose tolerance test (GTT) were elevated in the WD group compared to the control group, but were similar to normal values in the PNVs‐ and EA‐treated groups (Figure S3b). In addition, liver oxidative stress markers (e.g., CYP2E1, iNOS, and 3‐NT) and liver apoptosis markers (e.g., Bax, Cleaved caspase 3, P‐JNK) were respectively increased in the WDgroup, but their elevated levels were significantly decreased by the oral administration of PNVs or EA (Figure 4f,g). These results confirm that PNVs can prevent WD‐related MASLD by inhibiting elevated levels of liver oxidative stress and apoptosis marker proteins.

### 3.4 PNVs Attenuated the Increased Hepatic Fibrosis in MASLD Mice

**FIGURE 5:**
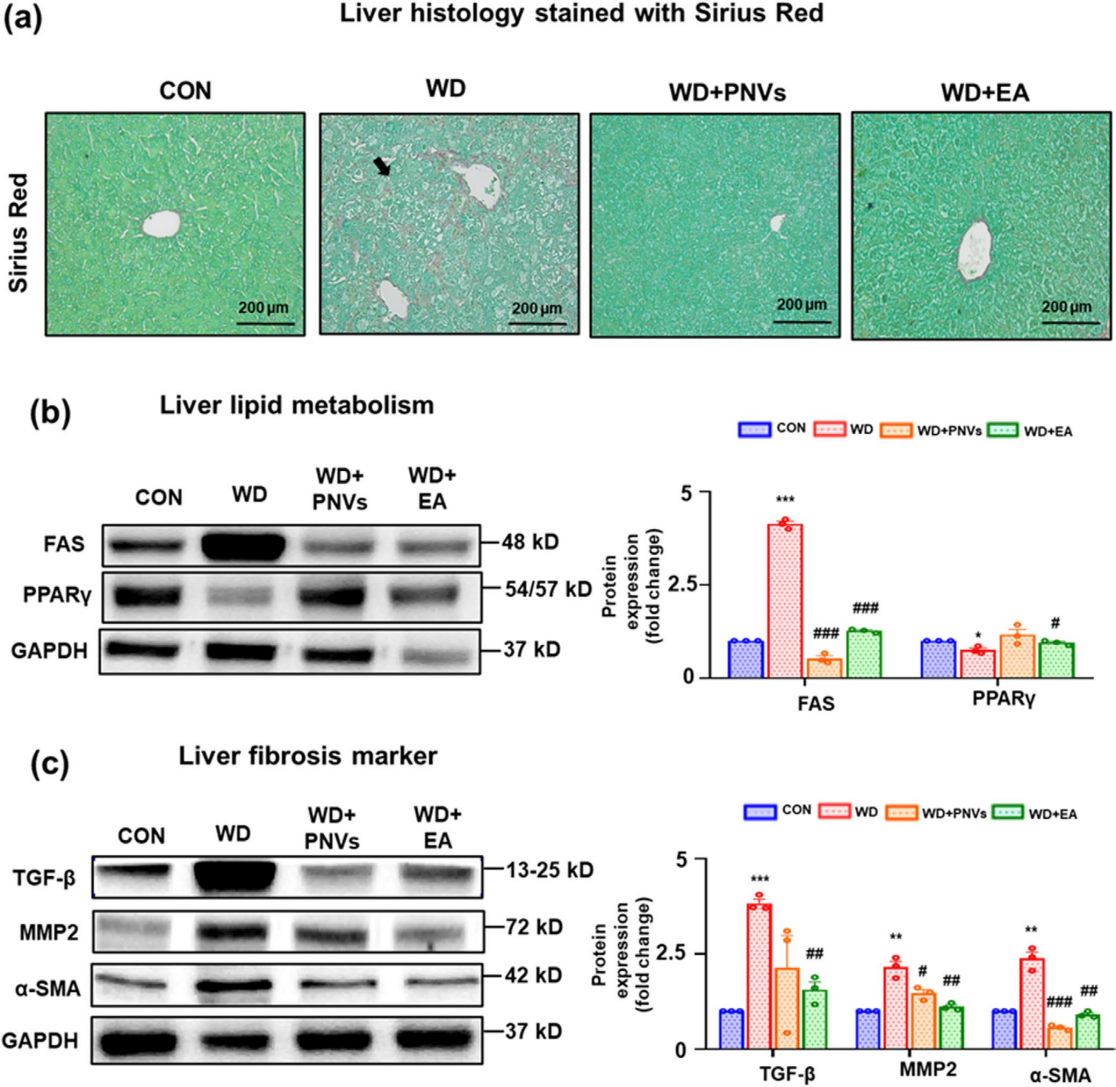
PNVs treatment attenuated WD‐induced liver fibrosis in MASLD mice. (a) As indicated, Representative Sirius Red‐stained liver sections for CON, WD, WD + 1 mg PNVs, and WD + 60 mg/kg EA. Immunoblot results for (b, c) the various liver lipid metabolism markers (FAS, PPARγ) and liver fibrosis markers (TGF‐ꞵ, MMP‐2, and α‐SMA) for the indicated groups. Densitometric analysis of the immunoblot results for each protein relative to the GAPDH loading control is shown. Each immunoblot experiment was repeated at least three times independently, and consistent results were observed. *p < 0.05, **p < 0.01, ***p < 0.001 between CON and WD groups; #*p* < 0.05, ##*p* < 0.01, ###*p* < 0.001 between WD and WD + 1 mg PNVs; #*p* < 0.05, ##*p* < 0.01, ###*p* < 0.001 between WD and WD + 60 mg/kg EA groups. The significance of mean values for each group was determined using the Student's *T*‐test.

The hepatoprotective effect of PNVs against liver fibrosis was evaluated in the MASLD mouse model. Sirius Red staining revealed increased areas of liver fibrosis in the WD group compared to the control group, suggesting that chronic exposure to the WD diet induced hepatic fibrosis in mice. However, WD‐mediated elevated fibrosis areas compared with the control group were significantly decreased in both PNVs‐ and EA‐treated groups (Figure 5a). In addition, liver lipid synthesis markers (FAS) and hepatic fibrosis markers (such as TGF‐ꞵ, α‐SMA, and MMP2) were increased in the WD‐treated group, but these elevations were significantly decreased by the oral administration of both PNVs and EA. Also, the levels of PPARγ were decreased in the WD‐treated group, but these changes were significantly increased by the oral administration of both PNVs and EA (Figure 5b,c). These results suggest that PNVs exhibit a beneficial effect on WD‐associated liver fibrosis by suppressing the increased levels of fibrosis markers.

### 3.5 PNVs Alleviated Changes in Gut Junctional Complex Proteins in MASLD Mice

**FIGURE 6:**
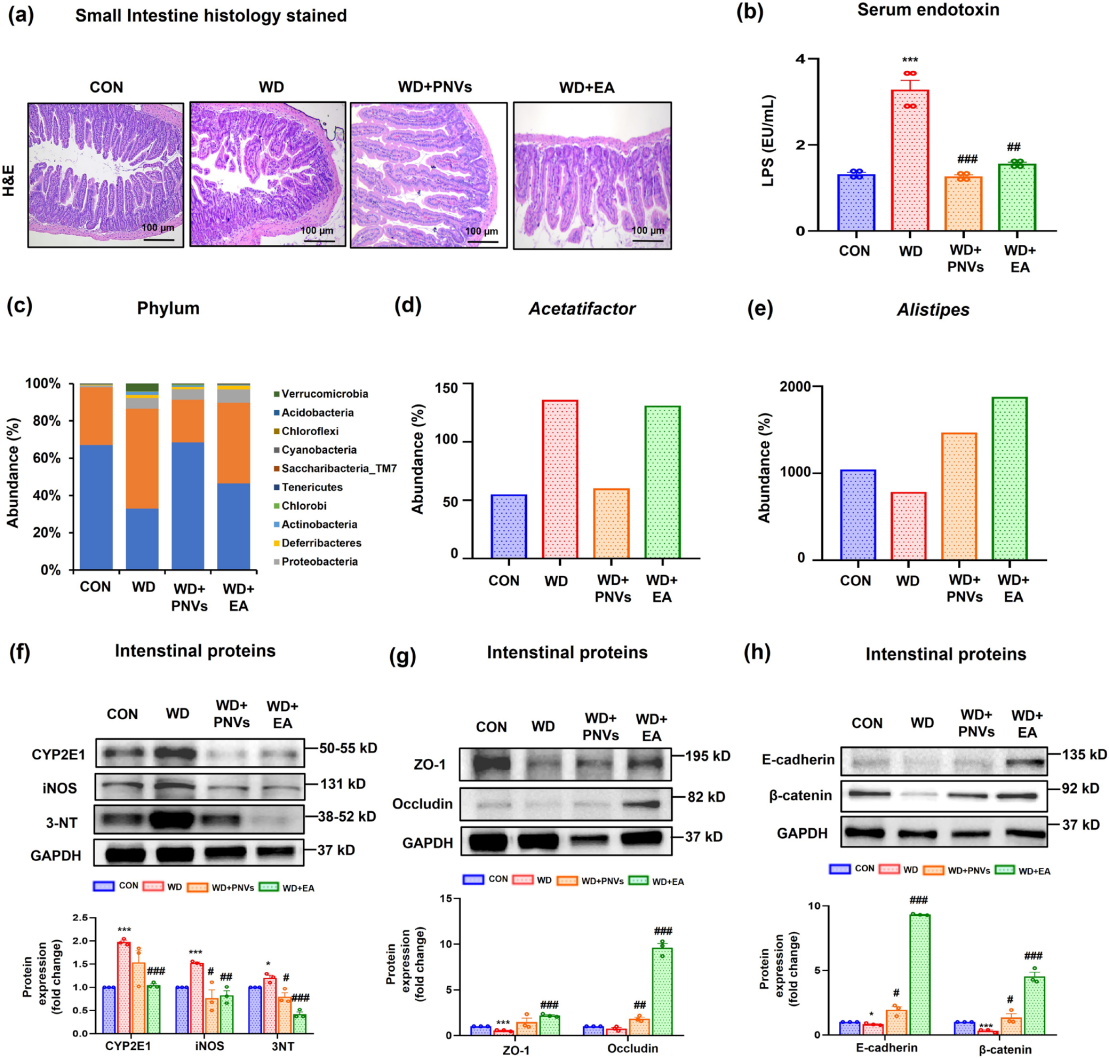
PNVs prevented WD‐induced leaky gut in MASLD mice. (a) As indicated, Representative H and E‐stained small intestine sections for CON, WD, and WD + 1 mg/kg PNVs, WD + 60 mg/kg EA. (b) The level of serum endotoxin. (c) Relative composition and abundance of various bacterial phyli in the overall gut microbiome for the three indicated groups. The relative abundances of genus *Acetatifactor* (d) or genus *Alistipes* (e) in the indicated groups are presented for comparison purposes. (f) The levels of oxidative stress protein markers (CYP2E1, iNOS, 3‐NT). (g) The levels of gut tight junction proteins (ZO‐1, Occludin), (h) Adherens junction proteins (E‐cadherin, β‐catenin). The densitometric result of immunoblot for each protein relative to the GAPDH loading control is shown. Each immunoblot experiment was repeated at least three times independently, and consistent results were observed. *p < 0.05, **p < 0.01, ***p < 0.001 between CON and WD groups; #*p* < 0.05, ##*p* < 0.01, ###*p* < 0.001 between WD and WD + 1 mg PNVs; #*p* < 0.05, ##*p* < 0.01, ###*p* < 0.001 between WD and WD + 60 mg/kg EA groups. The significance of mean values for each group was determined using Student's *T*‐test.

A recent study showed that intestinal microbial community imbalance plays an important role in non‐alcoholic fatty liver disease (NAFLD) by increasing intestinal permeability through dysregulation of the gut‐liver axis (Song and Zhang 2022). H and E‐stained histological analysis showed that administration of PNVs and EA improved abnormal gut villus structure caused by the WD diet (Figure 6a). In addition, the serum endotoxin (LPS) level was increased in the WD group, and this change was significantly alleviated by the administration of PNVs and EA, respectively (Figure 6b). The composition and abundance of the cecal microbiota were analyzed in the WD‐induced MASLD mice, with or without PNVs and EA treatment, and compared to the control group. Intestinal microbiome sequencing analysis showed a significant difference in phylum composition abundance among the control group, the WD mice, PNVs, and EA‐administered groups (Figure 6c). For instance, *Proteobacteria* (gray color) abundance was markedly increased in the WD group compared to the control but improved in the PNVs‐exposed group. *Bacteroidetes* (blue color) abundance was significantly decreased in the WD group compared to the control but recovered in the PNVs or EA‐exposed group. The abundances of genus *Acetatifactor* (Figure 6d) increased in the WD group but decreased in the PNVs‐administered group. In addition, the amounts of genus *Alistipes* (Figure 6e) decreased in the WD group but increased in the PNVs or EA‐administered group, as similar to the recent results (Xu et al. 2024). Also, the quantities of oxidative stress marker proteins (e.g., CYP2E1, iNOS, and 3‐NT) and junctional complex proteins (e.g., ZO‐1, Occludin, β‐catenin, and E‐cadherin) in the small intestine were significantly decreased in the WD group compared to the control group, but their levels were restored in the PNVs and EA‐treated groups (Figure 6f–h). Thus, administration of PNVs significantly reduced leaky gut and endotoxemia by elevating intestinal junctional complex proteins in MASLD mice, indicating the protective role of PNVs against MASLD‐associated gut injury as well.

## 4 Discussion

Edible plant‐derived exosome‐like nanovesicles (PENs) can be produced from various plants and fruits while they contain proteins, lipids, mRNA, miRNA, and numerous bioactive substances. PENs are natural nanocarriers with a very small size of 50–300 nm and are expected to be an effective nanomedical technology for disease prevention/treatment or drug delivery (Kim et al. 2022; Rahmati et al. 2024). Recently, our team demonstrated the efficacy of PENs using pomegranate, hemp, yam, and plum against various types of diseases, including ALD (Kim et al. 2023a, 2023b), anticancer (Kim et al. 2024), IBD (Eom et al. 2022), and osteoporosis (Hwang et al. 2023; Park et al. 2023). On the other hand, only one drug has been approved by the FDA to treat the chronic liver diseases MASLD and metabolic dysfunction‐associated steatohepatitis (MASH) and treatment options are very limited (Wang et al. 2024b). Therefore, the development of safe and effective drugs through PENs could become very important in treating or effectively managing the conditions of MASLD/MASH.

Pomegranates provide important therapeutic benefits for daily health care and disease prevention. Pomegranates are rich in anthocyanins, polyphenols, and ellagitannins (punicalagin, ellagic acid), which are known to inhibit oxidation and inflammatory processes. Especially, its major component EA has been proven to be further metabolized into urolithin by the gut microbiome, thereby alleviating oxidative stress, inflammation, and fibrosis associated with NAFLD (Senavirathna et al. 2024). Subsequently, many medicinal applications and pharmacological effects of pomegranate and EA have been reported (Li et al. 2023; Maphetu et al. 2022). Recent reports also showed that PNVs have the potential to treat ALD (Kim et al. 2023a, 2023b), NAFLD (Hou et al. 2023), anti‐cancer effects on breast cancer cells (Kim et al. 2024), and benign prostatic hyperplasia (Sreekumar et al. 2023) in experimental models. In this study, we assessed the effectiveness of PNVs in WD‐induced MASLD mice and explored their protective mechanisms, comparing them to EA alone, which served as the positive control.

PNVs were successfully isolated and purified using ultracentrifugation by following the previously established methods (Hwang et al. 2023; Kim et al. 2023a, 2023b). The morphological characteristics of PNVs were confirmed by TEM analysis. NTA was also performed to determine the size and distribution of PNVs, and that isolated PNVs had a small nano size and lipid layers. Leaky gut and microbiota dysfunction are important in the development and progression of NAFLD (Kobayashi et al. 2022). Gut microbiota was thought to be actively interacting with the dietary polyphenols, which influence the bioavailability of polyphenols from pomegranate (Cano et al. 2024; Duda‐Chodak et al. 2015; Mandal and Domb 2024). Animal models have also demonstrated that several factors contribute to and can significantly alter the composition and abundance of the gut microbiota. In this study, we showed that PNVs restore gut microbiota and alleviate liver fibrosis by improving gut leakiness and endotoxemia resulting from reduced intestinal junctional complex proteins. Our results showed that *Proteobacteria* (gray color) abundance was markedly increased in the WD group compared to the control but improved in the PNVs‐exposed group. *Bacteroidetes* (blue color) abundance was significantly decreased in the WD group compared to the control but recovered in the PNVs‐ or EA‐exposed group. Although we did not investigate the specific mechanism by which EA‐rich PNVs prevent WD‐mediated gut leakage, we analyzed the changes in the cecum microbiota, as the small intestine is considered an important site where the initial fermentation of polyphenols begins. In future studies, we plan to more comprehensively investigate the bioavailability of PNVs, identify different metabolites, and study the microbial communities in the colon. Our results suggest that PNVs contain substantial amounts of EA, which significantly prevents gut leakiness by upregulating intestinal junction proteins (ZO‐1, Occludin, E‐cadherin, β‐catenin) and inhibiting oxidative stress. Consistently, PNVs and EA significantly reduced the serum endotoxin levels in MASLD‐associated gut permeability, and these results were similar to those observed in our previous data using PENs isolated from other plants or EA alone, as reported by our laboratory (Kim et al. 2023a, 2023b, 2021). In fact, we previously reported that EA alone prevented gut permeability in the mouse models of both IBD and alcohol‐associated liver disease (Kim et al. 2023a, 2023b, 2021). Furthermore, PNVs and EA also prevented the MASLD‐induced increase in fat accumulation with elevated hepatic TG, liver lipid metabolism marker proteins such as FAS, PPARγ, and oxidative stress marker proteins such as CYP2E1, iNOS, and 3‐NT. In addition, PNVs and EA mitigated hepatic fibrosis, as indicated by Sirius red staining and the reduced levels of TGF, α‐SMA, and MMP2 in WD‐mediated MASLD. Additionally, ellagic acid (EA) contained in various plant‐derived PENs has been studied for its potential protective effects against liver fibrosis, demonstrating that their beneficial effects were similar to those of EA alone, which was used as a positive control in MASLD (Girish and Pradhan 2012; Thresiamma and Kuttan 1996; Zhao et al. 2021).

In summary, we have described the method to isolate PNVs and their beneficial effects against MASLD‐associated gut dysbiosis, intestinal permeability, fatty liver, and hepatic fibrosis. Previous studies have reported that various PENs have protective effects on liver diseases and metabolic diseases (Cho et al. 2018; Eom et al. 2022; Liu et al. 2020; Zhao et al. 2022). Despite these advantages, the limitations of this study can be that although EA was identified as the major bioactive component in PNV, our current study did not fully elucidate the beneficial effects of other components, such as different polyphenols, proteins, or nucleic acids. In addition, considering the complexity of human metabolic diseases, it is necessary to verify the therapeutic potentials of PNVs in humans with MASLD and compare them with the existing MASLD treatment drug(s). Overall, this study demonstrated that PNVs have inhibitory effects on intestinal leakage, fatty liver, and hepatic fibrosis. In the context of limited MASLD treatment options, PNVs can be considered as promising natural nanomedicines. Future studies should strengthen the mechanistic analysis and evaluate the possibility of clinical applications.

## 5 Conclusion

In conclusion, this study revealed the efficient isolation of exosome‐like nanovesicles extracted from pomegranate juice and the subsequent characterization of them using the standard methods. Interestingly, purified PNVs showed beneficial therapeutic potential in managing the MASLD model of intestinal and liver injury. In particular, PNVs rich in EA were shown to improve lipid accumulation and fibrosis in the liver by alleviating intestinal leakage caused by MASLD at least partly through the gut‐liver axis. Therefore, it is suggested that EA‐rich PNVs are promising therapeutic and protective agents with potential benefits not only in liver diseases but also in various other diseases through their antioxidant and anti‐inflammatory activities.

## Author Contributions

**Ji‐Su Kim:** conceptualization (equal), data curation (equal), investigation (equal), writing – original draft (equal), writing – review and editing (equal). **Byoung‐Joon Song:** writing – original draft (equal), writing – review and editing (equal). **Young‐Eun Cho:** conceptualization (lead), data curation (lead), investigation (lead), project administration (lead), writing – original draft (lead), writing – review and editing (lead).

## Conflicts of Interest

The authors declare no conflicts of interest.

## Supporting information


Data S1.

## Data Availability

All relevant data are within the manuscript and its Supporting Information.
