# Supplementary material for: Pomegranate‐Derived Exosome‐Like Nanovesicles Containing Ellagic Acid Alleviate Gut Leakage and Liver Injury in MASLD
Source: Food Sci Nutr. 2025 Apr 10;13(4):e70088. doi: 10.1002/fsn3.70088 (PMC11982932; doi:10.1002/fsn3.70088)
Supplement: Supplementary file 1 — Data S1. [file FSN3-13-e70088-s001.zip › FSN370088-sup-0002-Suppoting information.docx]

Supporting Information

**Pomegranate-derived exosome-like nanovesicles containing ellagic acid alleviate gut leakage and liver injury in MASLD**

Ji-Su Kim^1^*, Byoung-Joon Song^2,^ Young-Eun Cho^1^#

^1^Department of Food and Nutrition, Andong National University, Andong 36729, South Korea;

^2^ Section of Molecular Pharmacology and Toxicology, National Institute on Alcohol Abuse and Alcoholism, Bethesda, MD 20892, USA

#Corresponding author: Young-Eun Cho, Department of Food and Nutrition, Andong National University, Andong 36729, South Korea, E-mail: yecho@anu.ac.kr

**
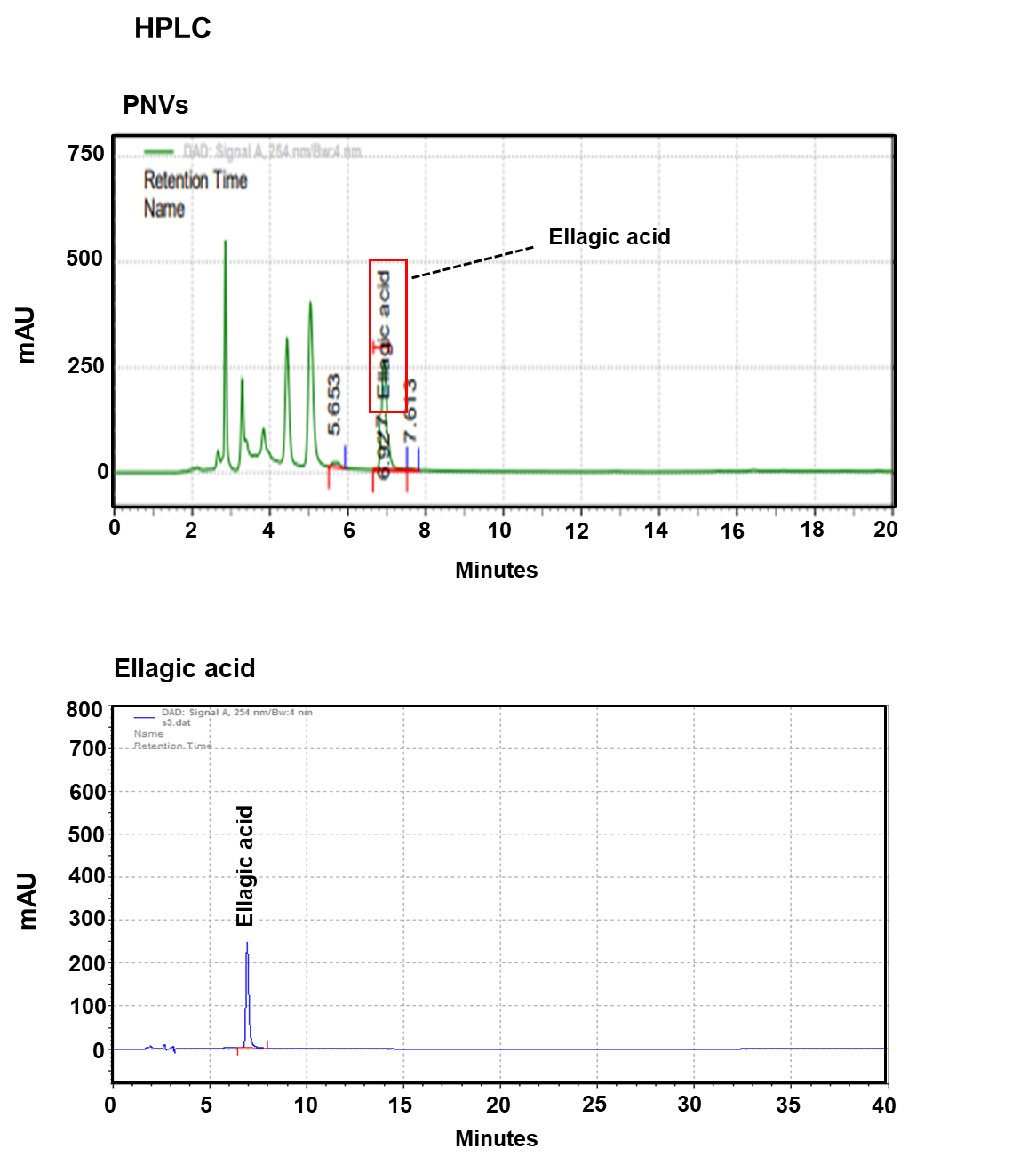
**

**Figure S1. HPLC chromatogram of PNVs**

HPLC chromatograms of PNVs components (top) and Ellagic acid (EA) standard (bottom), respectively


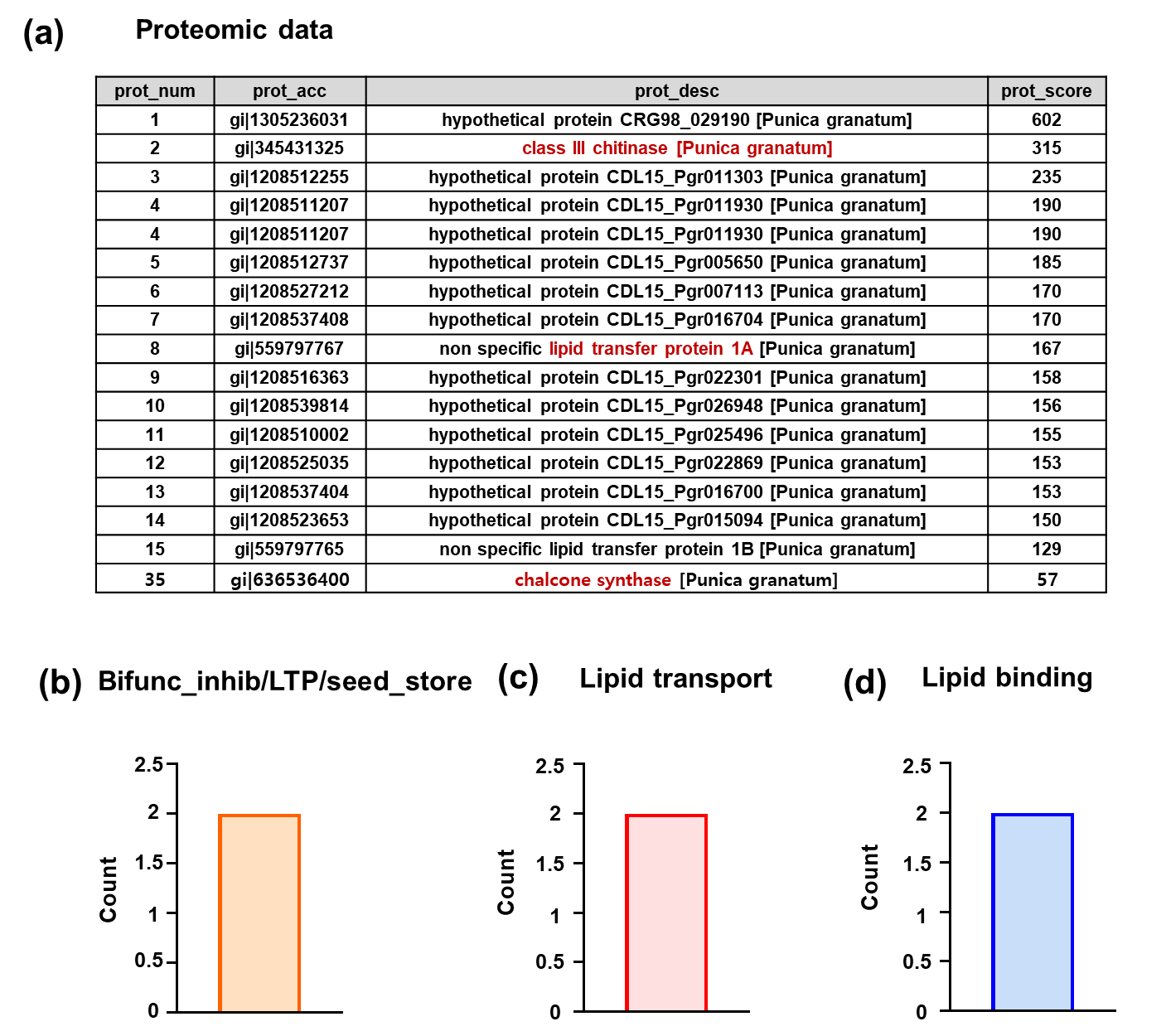


**Figure S2. Proteomic data of PNVs**

(a) Proteins present in the isolated PNVs are listed in the Table. These proteins are related to chitinase, lipid transfer protein, and chalcone synthase as presented in red color. (b-d) Gene ontology (GO) analysis show high levels of gene expression of bifunctional inhibitor/plant lipid transfer protein/seed storage helical (Bifunc_inhib/LTP/seed_store), lipid transport, and lipid binding function, as indicated.


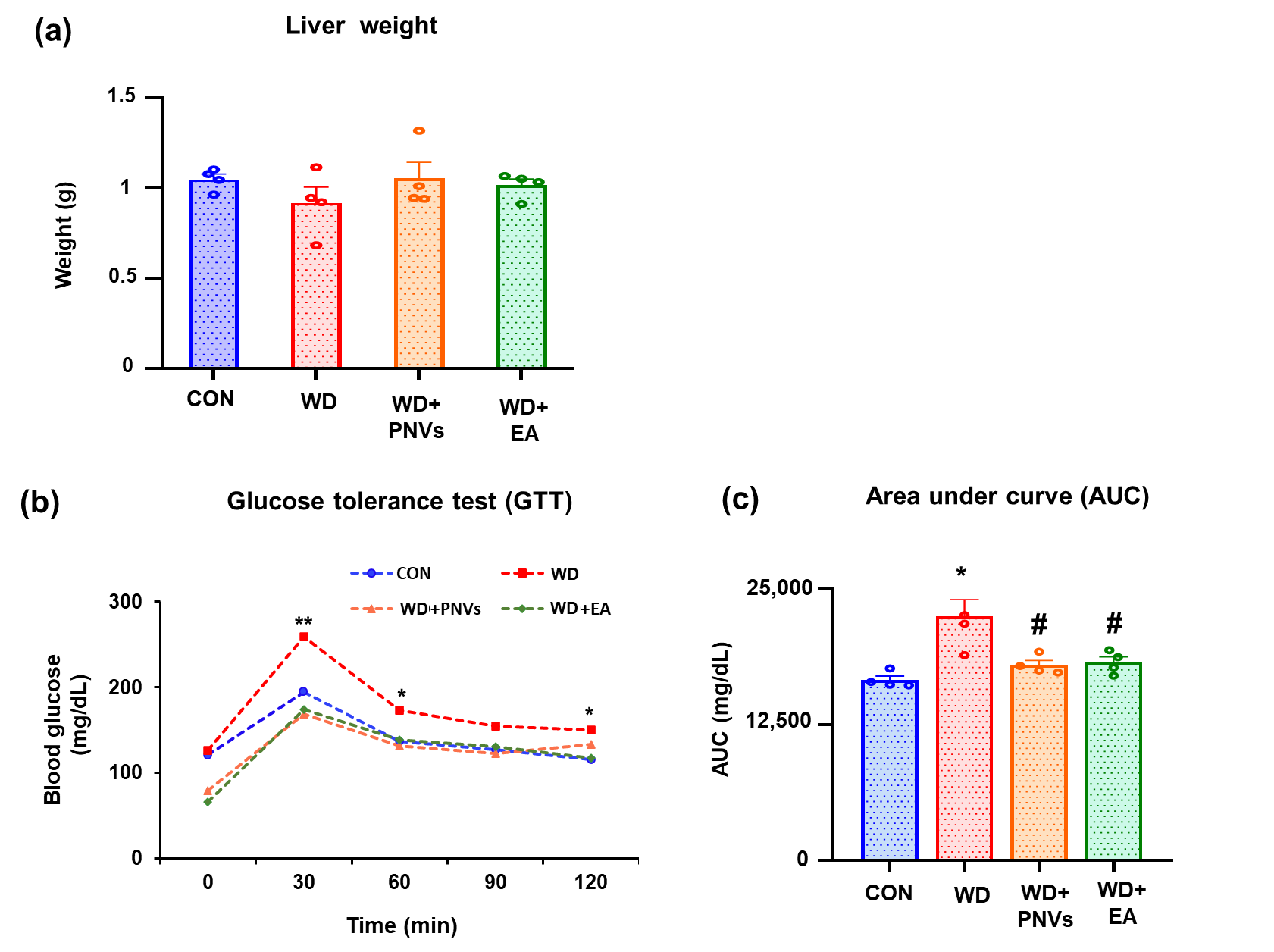


**Figure S3. In vivo liver weight and GTT results with or without orally administered PNVs**

(a) The average liver weight of the indicated mouse groups. (b-c) Impaired glucose metabolism determined by glucose tolerance test (GTT) and the area under the curve (AUC) were increased in the MASLD mice compared to the control group but restored by oral adminstration of PNVs or EA. *p<0.05, **p<0.01, ***p<0.001 between CON and WD groups; #p<0.05, ##p<0.01, ###p<0.001 between WD and WD+1 mg/kg/day PNVs; #p<0.05, ##p<0.01, ###p<0.001 between WD and WD+60 mg/kg/day EA group. The significance of mean values for each group was determined using Student’s T-test.

**Table S1. HPLC analysis conditions of EA.**

|  | **Column** | **Mobile phase** | **Flow rate (ml/min)** | **Temperature (°C)** | **Injection (μl)** | **UV (nm)** |
| --- | --- | --- | --- | --- | --- | --- |
| **EA** | C18  (4.6×250 mm，  5 μm)  (Agilent) | Acetonitrile-0.1% Formic acid | 1.0 | 40 | 5 | 254 |

**Table S2. List of the primary antibodies to each indicated protein used in immunoblotting analyses.**

|  | **Target protein** | **Dilution Factor** | **Source** | **Species** |
| --- | --- | --- | --- | --- |
| **Primary antibody** | **CYP2E1** | 1:5,000 | Abcam | Rabbit |
|  | **i NOS** | 1:5,000 | Abcam | Rabbit |
|  | **3-NT**  **FAS**  **PPARγ** | 1:5,000  1:1,000  1:1,000 | Abcam  Santa Cruz  Santa Cruz | Mouse  Mouse  Mouse |
|  | **α-SMA** | 1:5,000 | Sigma-Aldrich | Mouse |
|  | **MMP2**  **TGF-β** | 1:1,000  1:1,000 | Santa Cruz  Santa Cruz | Rabbit  Mouse |
|  | **ZO-1** | 1:5,000 | Abcam | Mouse |
|  | **β-catenin** | 1:1,000 | Santa Cruz | Mouse |
|  | **E-cadherin** | 1:1,000 | Santa Cruz | Mouse |
|  | **Bax**  **Occludin** | 1:1,000  1:1,000 | Santa Cruz  Santa Cruz | Mouse  Mouse |
|  | **Cleaved caspase 3** | 1:1,000 | Cell Signaling | Rabbit |
|  | **P-JNK** | 1:1,000 | Santa Cruz | Mouse |
|  | **JNK** | 1:1,000 | Santa Cruz | Mouse |
|  | **GAPDH** | 1:1,000 | Santa Cruz | Mouse |
