# Supplementary material for: Pomegranate‐Derived Exosome‐Like Nanovesicles Containing Ellagic Acid Alleviate Gut Leakage and Liver Injury in MASLD
Source: Food Sci Nutr. 2025 Apr 10;13(4):e70088. doi: 10.1002/fsn3.70088 (PMC11982932; doi:10.1002/fsn3.70088)
Supplement: Supplementary file 1 — Data S1. [file FSN3-13-e70088-s001.zip › Suppoting information.docx]

Supporting Table

**Table S1. HPLC analysis conditions of EA.**

|  | **Column** | **Mobile phase** | **Flow rate (ml/min)** | **Temperature (°C)** | **Injection (μl)** | **UV (nm)** |
| --- | --- | --- | --- | --- | --- | --- |
| **EA** | C18  (4.6×250 mm，  5 μm)  (Agilent) | Acetonitrile-0.1% Formic acid | 1.0 | 40 | 5 | 254 |

**Table S2. List of the primary antibodies to each indicated protein used in immunoblotting analyses.**

|  | **Target protein** | **Dilution Factor** | **Source** | **Species** |
| --- | --- | --- | --- | --- |
| **Primary antibody** | **CYP2E1** | 1:5,000 | Abcam | Rabbit |
|  | **i NOS** | 1:5,000 | Abcam | Rabbit |
|  | **3-NT**  **FAS**  **PPARγ** | 1:5,000  1:1,000  1:1,000 | Abcam  Santa Cruz  Santa Cruz | Mouse  Mouse  Mouse |
|  | **α-SMA** | 1:5,000 | Sigma-Aldrich | Mouse |
|  | **MMP2**  **TGF-β** | 1:1,000  1:1,000 | Santa Cruz  Santa Cruz | Rabbit  Mouse |
|  | **ZO-1** | 1:5,000 | Abcam | Mouse |
|  | **β-catenin** | 1:1,000 | Santa Cruz | Mouse |
|  | **E-cadherin** | 1:1,000 | Santa Cruz | Mouse |
|  | **Bax**  **Occludin** | 1:1,000  1:1,000 | Santa Cruz  Santa Cruz | Mouse  Mouse |
|  | **Cleaved caspase 3** | 1:1,000 | Cell Signaling | Rabbit |
|  | **P-JNK** | 1:1,000 | Santa Cruz | Mouse |
|  | **JNK** | 1:1,000 | Santa Cruz | Mouse |
|  | **GAPDH** | 1:1,000 | Santa Cruz | Mouse |
